# Rodent ultrasonic vocalizations are bound to active sniffing behavior

**DOI:** 10.3389/fnbeh.2014.00399

**Published:** 2014-11-18

**Authors:** Yevgeniy B. Sirotin, Martín Elias Costa, Diego A. Laplagne

**Affiliations:** ^1^Shelby White and Leon Levy Center for Brain, Mind and Behavior, The Rockefeller University, New YorkNY, USA; ^2^Integrative Neuroscience Lab, Department of Physics, University of Buenos AiresBuenos Aires, Argentina; ^3^Brain Institute, Federal University of Rio Grande do NorteNatal, Brazil

**Keywords:** ultrasonic vocalizations, rat, mouse, respiration, speech breathing, theta, rhythm, orofacial

## Abstract

During rodent active behavior, multiple orofacial sensorimotor behaviors, including sniffing and whisking, display rhythmicity in the theta range (~5–10 Hz). During specific behaviors, these rhythmic patterns interlock, such that execution of individual motor programs becomes dependent on the state of the others. Here we performed simultaneous recordings of the respiratory cycle and ultrasonic vocalization emission by adult rats and mice in social settings. We used automated analysis to examine the relationship between breathing patterns and vocalization over long time periods. Rat ultrasonic vocalizations (USVs, “50 kHz”) were emitted within stretches of active sniffing (5–10 Hz) and were largely absent during periods of passive breathing (1–4 Hz). Because ultrasound was tightly linked to the exhalation phase, the sniffing cycle segmented vocal production into discrete calls and imposed its theta rhythmicity on their timing. In turn, calls briefly prolonged exhalations, causing an immediate drop in sniffing rate. Similar results were obtained in mice. Our results show that ultrasonic vocalizations are an integral part of the rhythmic orofacial behavioral ensemble. This complex behavioral program is thus involved not only in active sensing but also in the temporal structuring of social communication signals. Many other social signals of mammals, including monkey calls and human speech, show structure in the theta range. Our work points to a mechanism for such structuring in rodent ultrasonic vocalizations.

## Introduction

Many behaviors are organized into repetitive cycles. In active rodents, orofacial sensorimotor behaviors like sniffing, whisking, and head movements are organized into cycles with a characteristic frequency in the theta range ~5–10 Hz (Welker, 1964; Macrides, 1975; Deschênes et al., 2012). The cyclical nature of these behaviors serves to structure both sensory input and motor output (Ganguly and Kleinfeld, 2004; Kepecs et al., 2006). However, while each behavior can independently display characteristic patterns, they often phase lock to each other (Welker, 1964; Moore et al., 2013; Ranade et al., 2013). This not only yields coordinated patterns of behavior, but also coordinated activity in associated neural circuits (Kay, 2005; Grosmaitre et al., 2007; Cury and Uchida, 2010; Shusterman et al., 2011; Deschênes et al., 2012; Miura et al., 2012; Moore et al., 2013). Indeed, both hippocampal and cortical theta rhythms can transiently phase lock to motor theta rhythms during specific behaviors (Komisaruk, 1970; Macrides et al., 1982; Ganguly and Kleinfeld, 2004; Kay, 2005; Shusterman et al., 2011). Such structuring suggests that our understanding of each individual behavior can benefit from consideration of the broader behavioral context.

The vocal behavior of rats and mice is proposed to feature two mechanisms of sound production. Audible vocal output of fundamental frequency below 20 kHz is produced, as in human speech, when air flowing out through tensed vocal folds causes them to vibrate resulting in sound pressure waves of rich harmonic content (Roberts, 1975a). Vocalization of fundamental frequency in the ultrasonic range (>20 kHz) is believed to be produced when air flowing through a small orifice formed by tight vocal folds produces ultrasound of nearly pure single frequencies via an aerodynamic whistle mechanism (Roberts, 1975b; Riede, 2011). Rat ultrasonic vocalization falls in two families with distinct ethological and neurophysiological parallels (Brudzynski, 2009). Aversive settings such as the anticipation of pain or danger can result in prolonged emission of ultrasound in the 20–25 kHz range with little or no frequency modulation, named “22 kHz” ultrasonic vocalizations (USVs). Ultrasound in the ~30–90 kHz range (“50 kHz USV”) is generally emitted by males and females in mating and other social interactions. Emission of 50 kHz USVs has been further linked to expectation of reward and activation of mesolimbic dopaminergic pathways (reviewed in Brudzynski, 2013). In turn, listening to 50 kHz USVs effectively induces approach behavior in both male and female rats, suggesting they may promote social contact (Wöhr and Schwarting, 2007; Seffer et al., 2014; Willadsen et al., 2014). Mice lack a 22 kHz-like alarm vocalization, and emit brief USVs in the ~50–100 kHz range, mostly studied in the context of mating (Holy and Guo, 2005). Vocalizations are usually segmented by experimenters into individual packets (“calls” or “syllables”) based on silences and/or spectral discontinuities (Liu et al., 2003; Wright et al., 2010). Interestingly, when segmenting by silences of 40 ms and over, adult rat and mouse calls are found to come in bouts with instantaneous rates in the theta range (Liu et al., 2003; Kim and Bao, 2009).

Vocal output depends critically on air flowing through the larynx, which is temporally structured by the breathing cycle (Roberts, 1975a). As in birds and humans, ultrasonic vocalizations in rats have been shown to be associated with increased subglottal pressure, indicating a phasic relationship with the breathing cycle (Roberts, 1972; Hegoburu et al., 2011; Riede, 2011, 2013). Highly vocal animals like humans and birds developed exquisite control mechanisms that coordinate breathing with activity in muscles used for vocalization in order to produce complex vocal output (MacLarnon and Hewitt, 1999; Andalman et al., 2011). As previously shown by us and others, rats show this control to some degree as they are able to maintain exhalations of over 2 s during the emission of prolonged 22 kHz alarm calls (Hegoburu et al., 2011; Assini et al., 2013). Rat breathing patterns are additionally constrained by sniffing, which is an active breathing behavior used to sample the olfactory environment (Welker, 1964; Wachowiak, 2011). Breathing patterns associated with normal respiration can be distinguished from active sniffing based on their frequency. Normal respiration in adult rats is typically below 3 Hz whereas active sniffing is typically in the theta range (Welker, 1964; Hegoburu et al., 2011; Wachowiak, 2011). However, despite clear dependence of vocalizations on breathing, the interplay between 50 kHz USVs and respiratory dynamics has not been previously investigated.

Here we examined, in detail, the relationship between respiration and ultrasonic vocal output of rats in a social environment. We find that ultrasonic vocalization of the 50 kHz family is largely restricted to periods of active sniffing (5–10 Hz). Within each sniff, both the initiation and cessation of vocal output was precisely linked to specific phases of the sniff, initiating just after the end of the inhalation and finishing just prior to the peak of the exhalation. As a result, the sniff cycle segments ultrasound production into individual calls, which inherit its theta rhythmicity. In turn, vocal output deforms ongoing sniff rhythms, briefly stretching the exhalation period as necessary to accommodate the full duration of the produced vocalization.

Our results show that orofacial behaviors with theta rhythmicity are not only involved in active sampling but also temporally structure outgoing communication signals at this rate. Moreover, we show that the sniffing and ultrasound production systems in rodents are linked on a millisecond scale, suggesting a tight coupling between the neural centers controlling sniffing and vocalizations.

## Materials and methods

### Animal subjects

All procedures were approved by The Rockefeller University Institutional Animal Care and Use Committee. Simultaneous recording of ultrasonic vocalizations and intranasal pressure were carried out on 5 Long Evans adult male rats (Charles River, ages 3–8 months, single housed from 2 months of age), and 2 CBA/CaJ adult male mice (Jackson Labs, ages 10–11 weeks, pair housed). Male mice were recorded in the presence of an adult female C57 mouse. Rats were held on an inverted light cycle and all recordings were carried out during the dark phase under infrared illumination.

### Recording sessions

**Figure 1:**
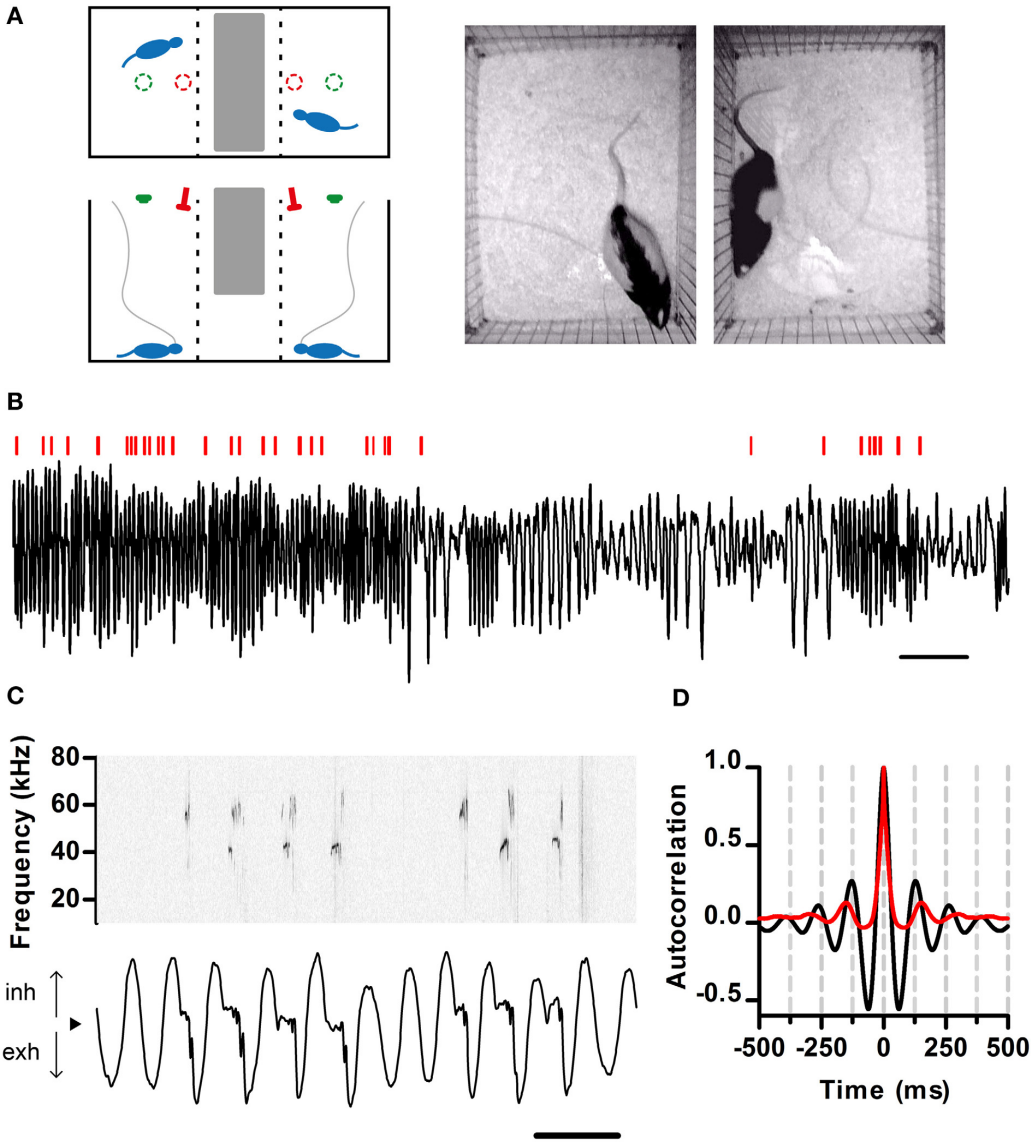
**Simultaneous recording of respiration and ultrasonic vocalization. (A)** Left: Schematic of the recording arena as viewed from the top (top) and side (bottom). The position of the ultrasonic microphones (red) and video cameras (green) is shown. Tubing (gray) connects the nasal cannulae with pressure sensors. Right: Snapshot of rats simultaneously behaving in the arena. **(B)** Segment of intranasal pressure (black) recorded from a rat in a social setting. Red bars: periods of ultrasonic vocal output detected for this rat. Scale bar: 2 s. From here on, inhalations are plotted as positive deflections of the pressure trace. **(C)** Detailed view of respiration (bottom) and ultrasonic vocalizations (top; sonogram). From here on, black arrowheads denote zero relative intranasal pressure. Scale bar: 250 ms. **(D)** Autocorrelations of respiration (black) and ultrasonic vocalizations (red) from a 10 min recording segment. Note signals show similar periodicity, with first peaks at 130 and 150 ms respectively (eq. 7.7 and 6.7 Hz). **(A–C)** same data set.

Rats were placed in a custom built social arena in a single-walled soundproof room. The purpose of this setup was to promote vocal production from social interaction while still being able to unequivocally assign each call to the rat it originated from. The arena (see Figure 1A) was split in two halves, 46 × 33 × 74 cm (W × L × H) each, 25 cm apart on the wide side. Walls were made of thin vertical bars and surrounded by 5 cm thick wedged foam to minimize echoes. The separation between halves was packed with foam from 20 cm above the floor to the top to minimize cross-talk between microphones (see below). The acrylic floor was covered with Aspen Chips bedding (NEPCO, Warrensburg, NY, USA), chosen to minimize locomotion related noise (the same bedding was used in the home cages). One rat was placed on each side of the arena where they could hear and smell each other for sessions lasting up to 2 h. Male-female mice pairs were recorded together in a 20 × 40 × 30 cm (W × L × H) acrylic box with Aspen Chips bedding. The respiration of the female mouse was not monitored. Intranasal pressure and ultrasound signals were simultaneously digitized by a data acquisition board at 250 kHz sampling frequency (PCIe-6259 DAQ with BNC-2110 connector, National Instruments). Animals were monitored from outside the room through video under infrared illumination.

### Ultrasonic vocalizations

#### Recording and detection

One condenser microphone with nearly flat (±5 dB) response from 10 to 150 kHz (CM16/CMPA-5V, Avisoft Bioacustics) was positioned above each rat at a height of 72 cm to selectively pick up calls from the rat beneath (Figure 1A). All USV analysis was performed on the raw sound recordings with custom built MATLAB routines (The Mathworks). To efficiently handle the large recorded datasets, we developed automated techniques for detecting ultrasound emissions and assigning them to the rat of origin (Figure S1). The performance of our detection and assignment methodology was assessed in an independent set of recordings (see below). We first obtain the sonogram for each microphone (Figure S1A, 2 ms time window, 0.25 ms time step, 1 kHz bandwidth, 3 tapers; http://chronux.org/; Mitra and Bokil, 2007). Each time step of the spectrogram constitutes a vector *P* where each point is the power at a given frequency (18–100 kHz). We next normalize this vector by its sum (to ensure all values span between 0 and 1) and calculate the entropy of this normalized vector *P**_n_* as *H* = −*P**_n_* · *log*_2_*P**_n_*. For rodent vocalizations, sound power is concentrated at a single frequency, reducing the entropy, while unwanted noise is typically broadband and thus of high entropy (Figure S1A). Segments lasting at least 3 ms with entropy below a fixed threshold of 6.5 bits and bounded by silences of >20 ms are selected as putative USVs. These are then curated by automatically discarding as noise those with high power in the sonic range (5–18 kHz) and visually inspecting those with intermediate levels of ultrasonic entropy and sonic power. In a dataset of 31 recording sessions we estimated 94% of emitted USVs (47866 of an estimated total of 51095) were effectively detected in this way (Figure S1B).

Detected USVs are assigned to the emitting rat by comparing the signals from both microphones. When ultrasound is detected (crosses the entropy threshold) at only one microphone, the USV is assigned to the rat on the same side of the arena. If the same USV is detected at both microphones, it is assigned to the rat under the microphone with lowest entropy (examples in Figure S1A). To assess the accuracy of the USV assignment we analyzed 11 recording sessions with just one rat in the arena. 77% of calls (20653 of 26815) were detected only by the microphone on the rat's side (Figure S1C). Of those detected in both, the entropy difference was large enough to unambiguously assign them to the correct side of the arena (Figure S1D). Overall, 99.8 ± 0.1% of USVs were properly assigned at each session. In the special case of two rats vocalizing at the same time, they will typically produce USVs with different fundamental frequency profiles at each microphone. When these profiles are found to differ by >1 kHz during >3 ms we deduce both rats vocalized simultaneously and assign to each the USV detected by the microphone on its side (Figure S1E).

Mice USVs were recorded from a single condenser microphone positioned 30 cm above the floor and detected in a similar fashion. As justified in section Structuring of Mouse Ultrasonic Vocalizations by Sniffing, all calls were assigned to the male mouse.

#### Analysis

“Vocal ratio” was defined as the fraction of time (0–1) spent producing ultrasound in a window of 3 s. This measurement is independent of any segmentation of vocal production. A “call” was defined as the ultrasound emitted within an individual sniff. “Call rate” as the number of detected calls per second in a 3 s window. “Instant call rate” was calculated for calls occurring on consecutive sniffs as the reciprocal of the time between the onsets of the two calls (**Figure 6D**).

### Sniffing

#### Cannula implantation

To monitor respiration, the end of a thin 2-cm-long stainless cannula (gage 22) was implanted in the nasal cavity. The cannula was bent to an S-shape so as to end above the temporal bone. Animals were anesthetized using isoflurane gas anesthesia. A skin incision was made exposing the frontal bone and most of the nasal bone. A small hole was drilled in either the left or the right nasal bone, into which the tip of the cannula was inserted from above so as to protrude into the nasal cavity. The cannula was affixed to the hole with a small drop of cyanoacrylate glue (All-purpose Krazy Glue), and stabilized on the skull with methyl methacrylate dental cement around skull screws. Animals were given at least 2 days after a surgery for recovery.

#### Data acquisition and pre-processing

During experiments, the cannula was connected to a pressure sensor located above the arena (24PCAFA6G, Honeywell; modified to reduce internal air volume) with ~100 cm of Teflon tubing (AWG# 22 STD, Pennsylvania Fluorocarbon) via a plastic fluid swivel (375/22PS, Instech). The output of the pressure sensor bridge was coupled to an instrumentation amplifier (AD620, Analog Devices) for recording. For analysis, signals were downsampled to 1 kHz Inhalations caused an inward flow of air through the nose that resulted in a decrease in measured pressure whereas exhalations caused an outward flow of air through the nose resulting in an increase in the measured pressure signal. Throughout the figures, inhalations are shown as upward deflections and zero denotes atmospheric pressure.

The tubing connecting the cannula to the pressure sensor filters down fast fluctuations and imposes a time delay to the pressure signal. To measure this distortion we generated broadband pressure signals with an electrodynamic transducer (ET-132-203; Labworks Inc.) driven by a linear power amplifier (PA119; Labworks Inc.). We then recorded the same signal with our pressure sensor directly at the output of the transducer and after distortion by the tubing (Figure S2A). We used these two signals to calculate the transfer function of the tubing through Fourier deconvolution (http://terpconnect.umd.edu/~toh/spectrum/Deconvolution.html) and used this transfer function to reconstruct the undistorted intranasal pressure signal in all recordings (see Figure S2 for validation).

#### Analysis

To identify individual respiratory cycles (“sniffs”), we developed MATLAB routines to segment the recorded pressure traces as follows. Slow drifts in sensor output were removed (400 Hz low pass Butterworth filter). Signals were then mean subtracted and divided by their standard deviation. Sniff cycles were defined to start at the inhalation onset and end at the exhalation offset (onset of the next inhalation). Inhalation onsets were detected as positive slope crossings of a fixed threshold. The end of each inhalation was defined as the negative slope crossing of the same threshold. Sniffs with aberrant inhalation durations (<20 ms) were rejected from subsequent analyses.

The phase within the sniffing cycle was computed using a previously described algorithm (Shusterman et al., 2011). Briefly, we determined three points in time for each cycle: inhalation onset, inhalation offset (exhalation onset), and exhalation offset, as described above. We then morphed each sniff cycle so that the duration of its inhalation and exhalation matched the average durations across all recorded sniffs. Phase within the sniff was then defined as the normalized time (0–1) within the morphed sniff (see Figures 1A,B in Shusterman et al., 2011).

The instant rate of a sniff cycle was defined as the reciprocal of the time between the start of its inhalation and that of the next cycle. “Ongoing sniff rate” is calculated as the mean instant rate in 3 s windows. Only silent sniffs were included to specifically quantify the respiratory rhythm without direct effects from USVs (see **Figures 6A,D**).

### Bout analysis

For the analysis of call bouts, a binary vector was constructed for each recording session. Each vector element corresponded to a single sniff and was assigned 1 if the sniff was vocal and 0 if the sniff was silent. A call bout was defined as a stretch of calls occurring over consecutive sniff cycles (a stretch of ones in the vector). Distributions of bout lengths were obtained by pooling across sessions for each rat. Two random models were used to generate surrogate binary vectors. First, we constructed a constant probability model, where a single call probability was used for each vector element (i.e., sniff). Each sniff was randomly assigned a call with a fixed probability obtained by dividing the total number of calls over the total number of sniffs. For the variable probability model, we simulated the effect of a varying call production rate within a session. The probability of assigning a call to each surrogate element was obtained from the measured data as follows. We convolved the observed binary vector with a Gaussian kernel to estimate an underlying local call production probability. In this analysis, “rate estimation window” corresponds to the full width at half maximum of this kernel (measured in number of sniffs). To capture potential call probability fluctuations at different time scales, we generated surrogate datasets with models of different rate estimation window from 4 to 256 sniffs. For each session and model, we generated 1000 pseudorandom surrogate vectors, calculating the distribution of bout lengths for each. For each session, we calculated the log likelihood of observing a given bout length in the real vs. surrogate data as log_10_ of the ratio between the probability of observing a bout of a given length in the real data and that of the surrogates. For example, a value of 1 is obtained if a given bout length is 10 times more likely in the real data.

### Statistical analysis

**Figure 2:**
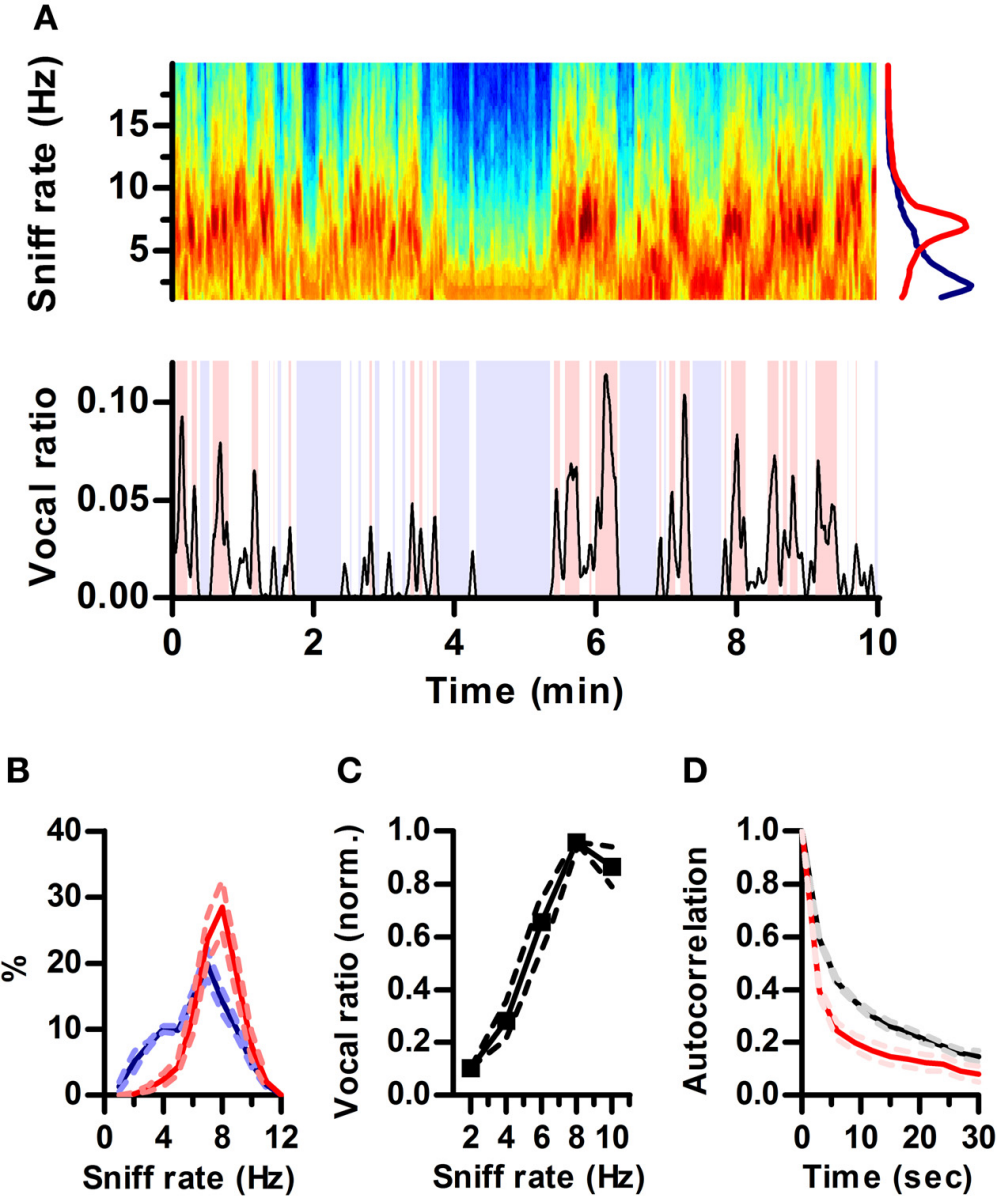
**Ultrasonic vocalization occurs during periods of fast sniffing. (A)** Top: Spectrogram of a section of the recorded respiration. Warmer tones denote higher power (AU). Note the alternation between periods of fast (~7 Hz) and slow (~2 Hz) respiration. Bottom: simultaneous vocal production from this rat quantified as fraction of time spent vocalizing within a 3 s sliding window (vocal ratio). Blue shading: periods of silence (vocal ratio = 0). Red shading: high vocal production (vocal ratio > 0.025). Top right: mean frequency spectrum of respiration for periods of high vocal production (red; peak = 6.8 Hz) and silence (blue; peak = 2.2 Hz) in the example. **(B)** Distribution of sniff rates during periods of high vocal production (red) or silence (blue). Mean ± s.e.m., *N* = 5 rats. **(C)** Vocal ratio as a function of sniff rate. To account for varying average vocal output of individual rats, curves were normalized by their maximum prior to averaging. Effect of sniff rate on mean vocal ratio: *p* < 0.0001 (ANOVA, *N* = 5 rats). **(D)** Autocorrelations of vocal ratio (red) and sniff rate (black), averaged in 3 s intervals.

**Figure 3:**
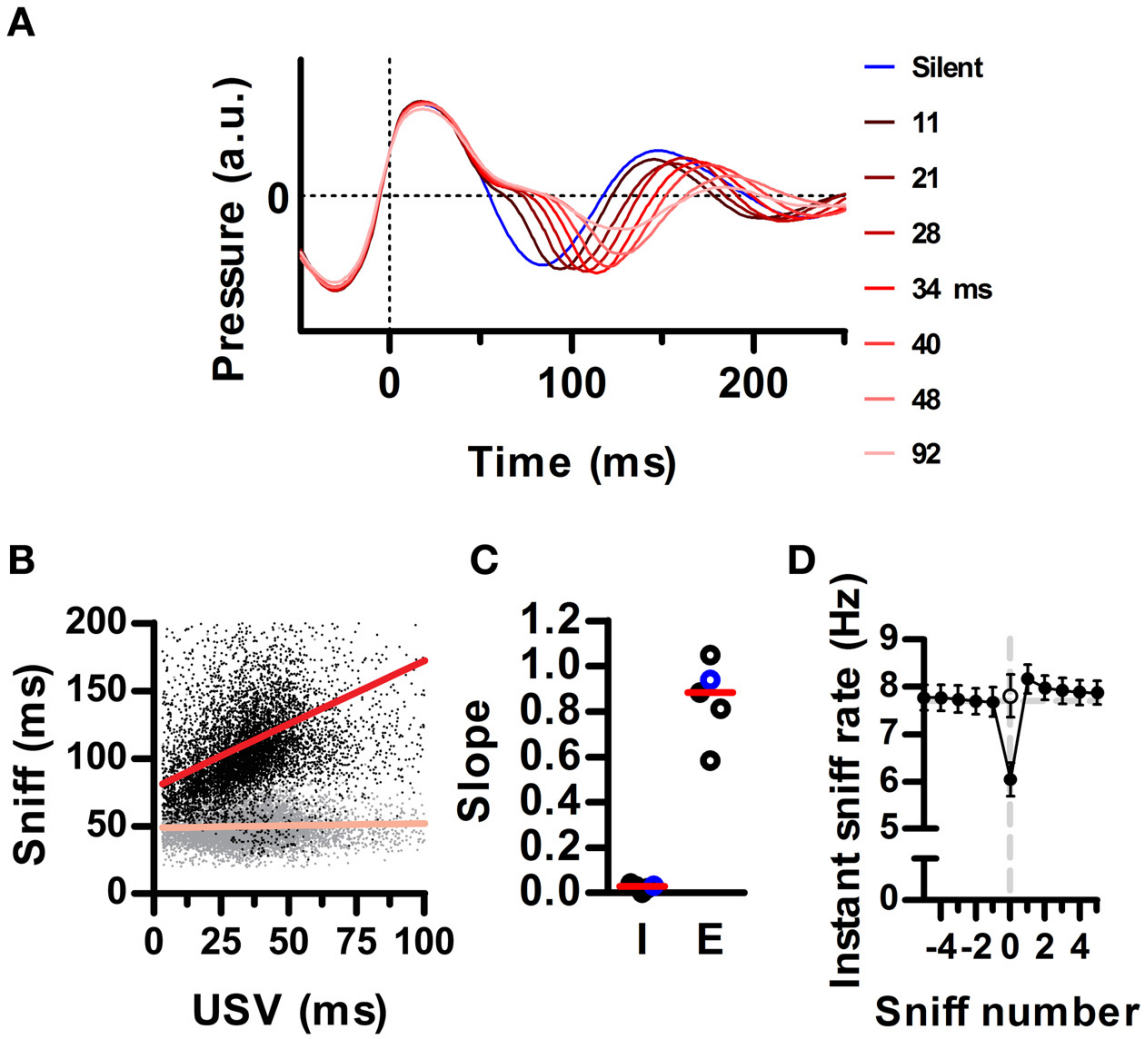
**Ultrasound emission instantaneously lowers the sniffing rate. (A)** Average waveforms for silent sniff cycles (blue) or cycles simultaneous to the emission of ultrasonic vocalizations of increasing duration (reds; vocal sniffs) for one example recording. Data was binned by ultrasound duration with bin centers labeled on the right. Traces are aligned to the onset of the inhalation (Time = 0). **(B)** Inhalation (gray) and exhalation (black) durations for individual vocal sniff cycles vs. vocalization duration. Same data as in A. Lines: linear regressions; *R*^2^ = 0.34 (exh) and 0.01 (inh). **(C)** Slopes for inhalation (“I”) and exhalation (“E”) regression lines for individual rats. Red lines: medians across animals. Values from B highlighted in blue. **(D)** Mean instantaneous sniff rate (1/sniff duration) aligned on a vocal sniff (sniff number = 0). Calculation of sniff rate for non-zero sniff numbers excludes vocal sniffs. Instantaneous sniff rate of vocal sniffs is lower than that of the preceding silent sniff (*p* < 0.001, paired *t*-test). Open circle: sniff rate computed after subtracting vocalization duration from the period of the vocal sniff. (Means ± s.e.m., *N* = 5 rats).

Relationships showing apparent linearity were analyzed with linear regression (**Figures 3B, 6E,F, 7B**). Others with repeated measures ANOVA (Figures 2C, **6B,C**).

## Results

To examine the relationship between respiration dynamics and ultrasonic vocal output of rats, we developed a split social arena. In the arena, adult male rats separated by a wire divider could hear and smell each other in the dark (Figure 1A). Analysis of audio from a pair of overhead microphones allowed us to unequivocally assign vocalizations to each rat. To monitor respiration, we implanted the rats with intranasal cannulae coupled to pressure sensors (see Materials and Methods). We recorded respiration and vocalizations for extended periods of time (30–120 min) at high sampling frequency (250 kHz), which allowed us to examine relationships between these behaviors across multiple timescales (Figure 1). Rats showed large variations in the rate of respiration and ultrasonic vocalization (Figure 1B). Under these conditions, all vocal output was restricted to USVs of the 50 kHz family (Figure 1C). As expected, intranasal pressure traces showed strong periodicity in the theta range imposed by the inhalation-exhalation cycle. Interestingly, vocal output was also periodic at theta (Figure 1D).

### Rats produce ultrasound during fast sniffing

Respiration rate in awake rats varies with behavioral state over a wide range (1–10 Hz) (Wachowiak, 2011). In our recordings, rats also alternated between periods of silence and high vocal production (Figure 2A). Visual inspection of respiration and vocalization records suggested that rats vocalized mostly during periods of active sniffing (e.g., Figure 1B). To quantify this relationship, we computed “vocal ratio” as the fraction of time spent producing ultrasound in a sliding window of 3 s (Figure 2A bottom; Methods). We calculated average ongoing sniff rate in this same window by segmenting the continuous intranasal pressure traces into individual sniff cycles (sniffs) and computing their average instantaneous rate (Methods). To avoid possible interactions between ultrasound production and sniffing, we excluded sniff cycles associated with vocal production from the calculation of sniff rate. During silent periods (vocal ratio = 0), rats were either breathing passively (rate < 4 Hz) or actively sniffing (rate > 5 Hz), spending similar periods of time in each mode. In contrast, periods of high vocal output (vocal ratio > 0.025) were exclusively associated with active sniffing (Figure 2B). Overall, this results in a strong positive correlation between vocal production and ongoing sniff rate with maximal vocal output during periods of 8 Hz sniffing (Figure 2C). Changes in vocal ratio were, however, faster than those of respiratory rate (Figure 2D), reflecting that brief periods of high vocal production occurred within longer periods of fast sniffing (e.g., Figure 2A).

### Ultrasound production prolongs the sniff cycle

Mammalian vocalization usually prolongs the respiratory cycle (Smotherman et al., 2010). We analyzed whether this is also the case for the brief rat vocalizations of the 50 kHz family. During silent respiration, recorded intranasal pressure typically followed a sinusoidal pattern, indicating roughly equal time spent inhaling and exhaling (e.g., Figure 1, blue trace in Figure 3A). Of our full population of recorded sniffs (*N* = 256991 sniffs in 5 rats), vocal sniffs accounted for 15 percent (*N* = 37593). Despite our observation that ultrasound is produced during periods of high ongoing sniff rate, vocal sniffs were on average longer than silent sniffs (163 ± 64 vs. 131 ± 55 ms; median ± inter-quartile-range; *p* ≅ 0, Wilcoxon rank sum test for equal medians). Within each vocal sniff, we quantified the total duration of ultrasound production as the difference between the first and last time-point having ultrasound. We found that overall sniff length increased with ultrasound duration (Figure 3A). Specifically, it was exhalation durations that increased, while inhalations remained largely unaltered (Figure 3B). Exhalations grew with ultrasound duration with a mean linear slope of 0.85 (Figure 3C). As a consequence, the emission of ultrasound during a given sniff cycle was accompanied by an instantaneous drop in the sniffing rate (Figure 3D).

### Ultrasonic vocalization occurs at specific phases of the sniff cycle

**Figure 4:**
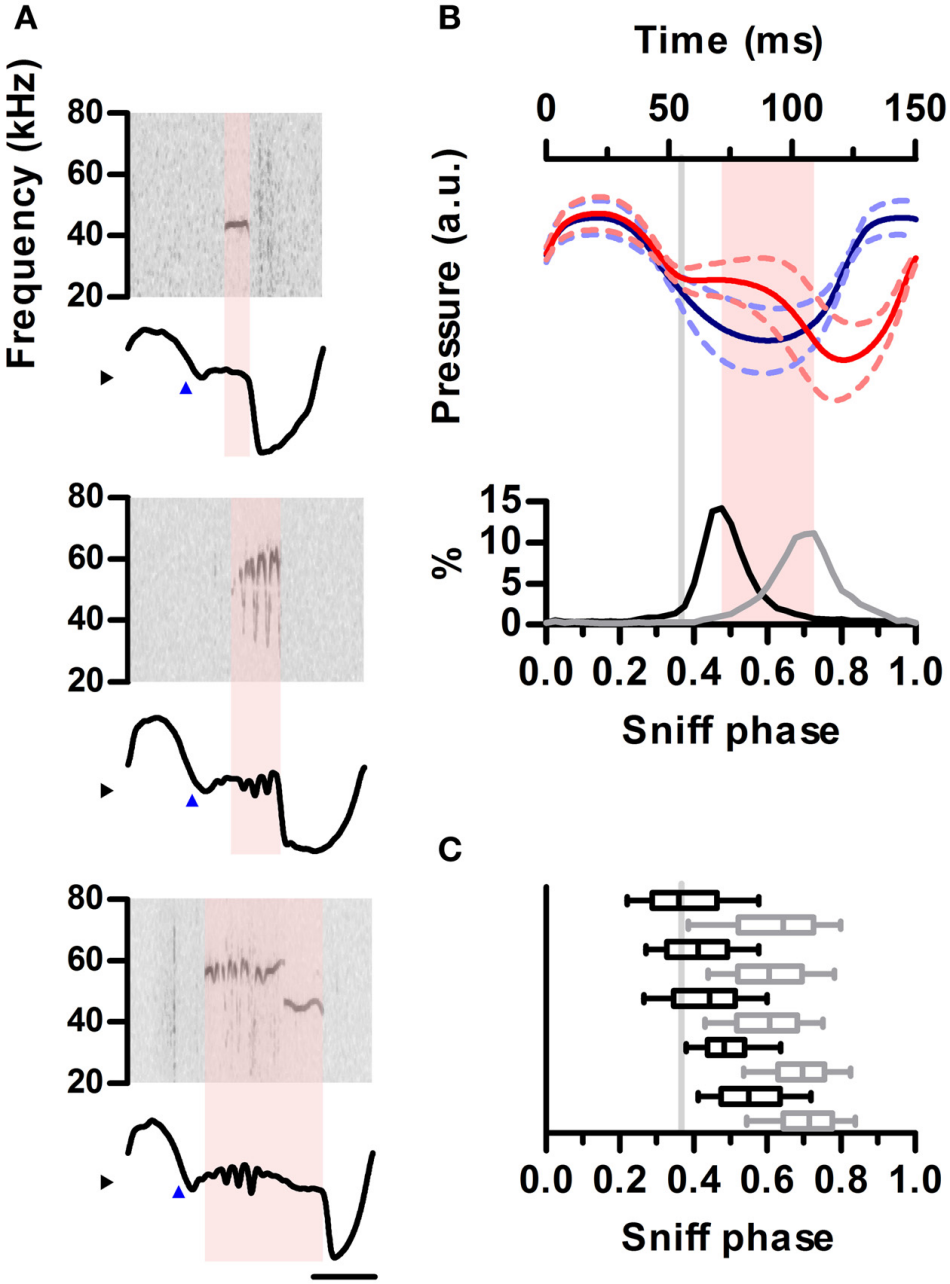
**Ultrasound emission is restricted to specific phases of the sniff cycle. (A)** Spectrograms from 3 ultrasound emissions (top) aligned to their corresponding intranasal pressure traces (bottom). Blue arrowheads mark exhalation onset. Note ultrasound is produced during low-pressure region following exhalation onset. **(B)** Top: mean sniff waveforms from silent (blue) or vocal (red) sniffs for one example rat. All waveforms were warped to align at three points: onsets of inhalation and exhalation and the end of exhalation. Bottom: distribution of ultrasound onset (black) and offset (gray) phases in the vocal sniffs. Inhalation onset: phase = 0, exhalation onset: phase = 0.33, end of exhalation: phase = 1. Gray line: exhalation onset. Time between most frequent vocalization onset and offset marked in pink. **(C)** Distribution of ultrasound onset (black) and offset (gray) phases for all rats. Boxes: median and 25–75th percentiles. Whiskers: 10–90th percentiles.

We next examined the detailed temporal alignment between ultrasound production and the inhalation-exhalation cycle. Prior work established that ultrasound is produced during exhalations, corresponding to periods of high subglottal pressure (Riede, 2011). Interestingly, during production of ultrasound, relative intranasal pressure remained close to zero, indicating reduced airflow through the nose (Figure 4A). This relationship held up to the millisecond timescale as brief drops in the power of the emitted ultrasound co-occurred with sharp peaks in nasal flow (Figure S3). We examined the coupling of ultrasound production to inhalations and exhalations by warping each sniff to a common phase axis aligning inhalation onsets, inhalation-exhalation transitions, and exhalation offsets (Methods). The average vocal sniff had a distinctly different shape than a silent sniff, with a pronounced deviation from a sinusoid after inhalation corresponding to the period of low airflow through the nose (Figure 4B, top). Indeed these shape differences were so pronounced that sniff shape alone was often an excellent predictor of the presence of vocalization (Figure S4). For all vocal sniffs, ultrasound production onsets and offsets were tightly coupled to sniff phase. Ultrasound production began shortly after the end of inhalation and ended prior to the peak of exhalation (Figure 4B, bottom). This tight coupling was observed in each of our tested animals (Figure 4C).

### The sniff cycle naturally segments emitted ultrasound into calls

**Figure 5:**
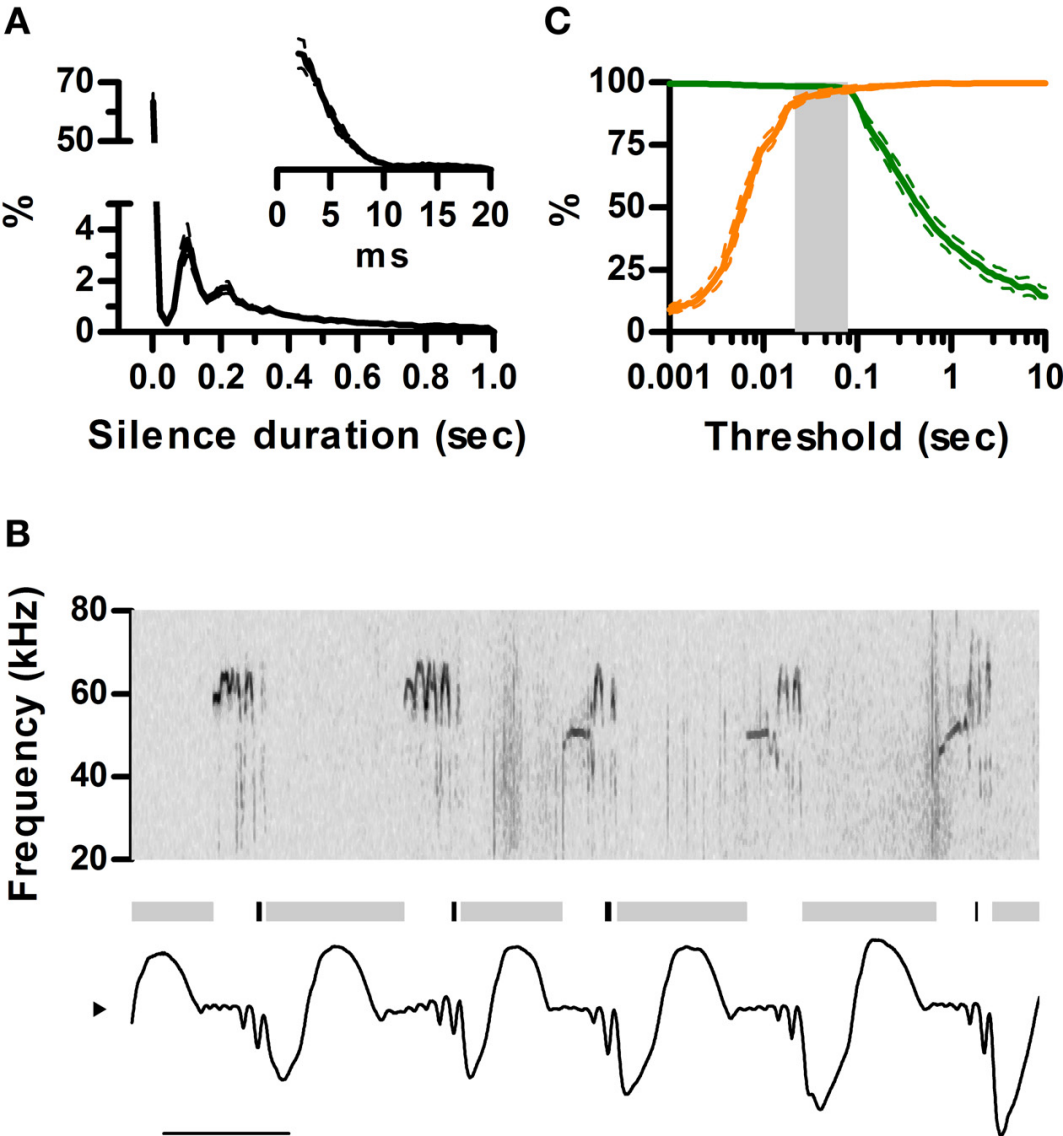
**Sniff cycles segment ultrasonic vocalization into calls. (A)** Distribution of silence durations (Mean ± s.e.m.; *N* = 5 rats). Inset: detail of short silences. **(B)** Example of ultrasonic vocalizations (top) and simultaneous sniffing (bottom). Gray and black bars mark the occurrence of long (>40 ms) and short (<40 ms) silences. Note short silences are contained within single exhalations while long ones span more than one sniff cycle. Scale bar: 100 ms. **(C)** Segmentation of calls as a function of silence duration threshold. Orange: Percentage of segmented calls that do not share a sniff cycle with other calls. Green: Percentage of calls that do not span more than one sniff cycle. The gray area shows the range of silence duration thresholds that effectively segment over 95% of calls by sniff cycles (20–80 ms).

Ultrasound appears to be emitted in brief units separated by silences, usually named “calls” or “syllables.” A clear rationale for this segmentation is, however, missing. It is clear from our data that rats are silent during inhalations. To understand how this structures the emission of ultrasound in time, we quantified the distribution of silence durations and its relation to the sniff cycle. We defined silences as intervals longer than 2 ms with no detectable vocal output. The analysis revealed identical multimodal distributions for all rats (Figure 5A). Silences were either shorter than 20 ms (58 ± 3%) or longer than 60 ms (41 ± 3%). Short silences occurred between ultrasound emissions within a single sniff cycle whereas long silences included at least one inhalation and thus separated emissions across sniffs (Figure 5B). In consequence, segmenting calls by a minimum silence of 20–60 ms is equivalent to segmenting by sniff cycle as all calls are moored to a single sniff and each sniff harbors at most one call (Figure 5C). The sniff cycle thus provides a natural segmentation of ultrasound production into individual calls.

### Ongoing sniff rate modulates call dynamics

**Figure 6:**
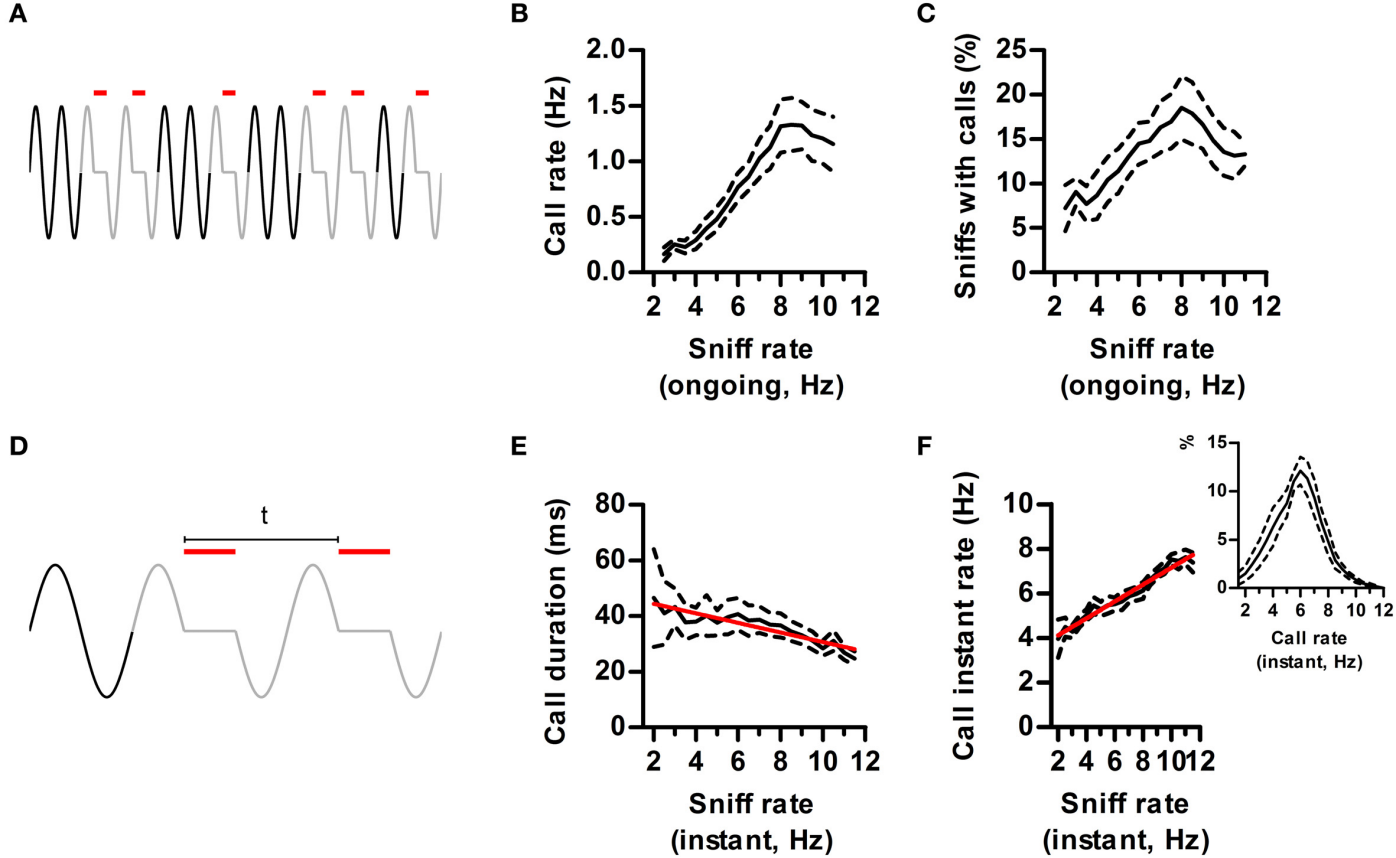
**Effects of sniff rate on call dynamics. (A)** Call rate is defined as the number of calls (red lines) in a time window of 3 s. Ongoing sniff rate is defined as the mean instant rate (1/sniff duration) of all sniffs with no USV (black sniffs) in the same window. **(B)** Call rate vs. ongoing sniff rate. Effect of sniff rate *p* < 0.0001, repeated measures ANOVA, *N* = 5 rats. **(C)** Percentage of sniffs accompanied by calls vs. ongoing sniff rate. Effect of sniff rate *p* < 0.0001, repeated measures ANOVA, *N* = 5 rats. **(D)** Instant call rate is defined as 1 over the time between the onsets of calls in two consecutive sniffs (“t” in figure). Instant sniff rate is that of the immediately preceding silent sniff. **(E)** Call duration vs. instant sniff rate. Red: linear regression; *R*^2^ = 0.12, *p* < 0.001. **(F)** Instant call rate vs. instant sniff rate. *R*^2^ = 0.67, *p* < 0.0001. Inset: distribution of instant call rates.

Studies on USVs typically correlate measurements like call rate and duration with experimental conditions. Having now defined a “call,” we analyzed to what extent their properties depend on the ongoing respiratory rate, assessed in neighboring silent sniffs (Figure 6A). As expected from our previous results, ongoing sniff rate strongly influenced measured call rates, which were maximal when sniffing at theta frequency (Figure 6B). The probability of emitting a call on each sniff also peaked during theta sniffing demonstrating that increased call rates were not trivially due to having more sniffs per unit time (Figure 6C).

So far we showed that sniff frequency strongly alters the quantity of calls produced. Does sniffing also alter the detailed dynamics of call production (Figure 6D)? We found that calls had a characteristic duration that was largely independent of sniff rate up to 8 Hz sniffing. However, for faster rates mean duration dropped by 25%, highlighting an interaction between the ongoing sniffing behavior and the vocal motor plan (Figure 6E).

We studied call rates in finer temporal detail by measuring the instant rate between calls occurring in consecutive sniffs (Figure 6D). As previously observed (Kim and Bao, 2009), rat calls have a characteristic instant rate of ~6 Hz (Figure 6F, inset). If this was a fixed property of USV emission mechanisms, instant call rate should be largely independent of ongoing respiratory rates. On the contrary, it was positively correlated to the rate of the immediately preceding silent sniff (Figure 6F). Thus, instant call rates carry information about ongoing sniffing frequency. This interaction is bidirectional, as calling immediately affects respiratory rate, bringing it to a narrower range centered at 6 Hz (Figure 6F).

### Structuring of mouse ultrasonic vocalizations by sniffing

**Figure 7:**
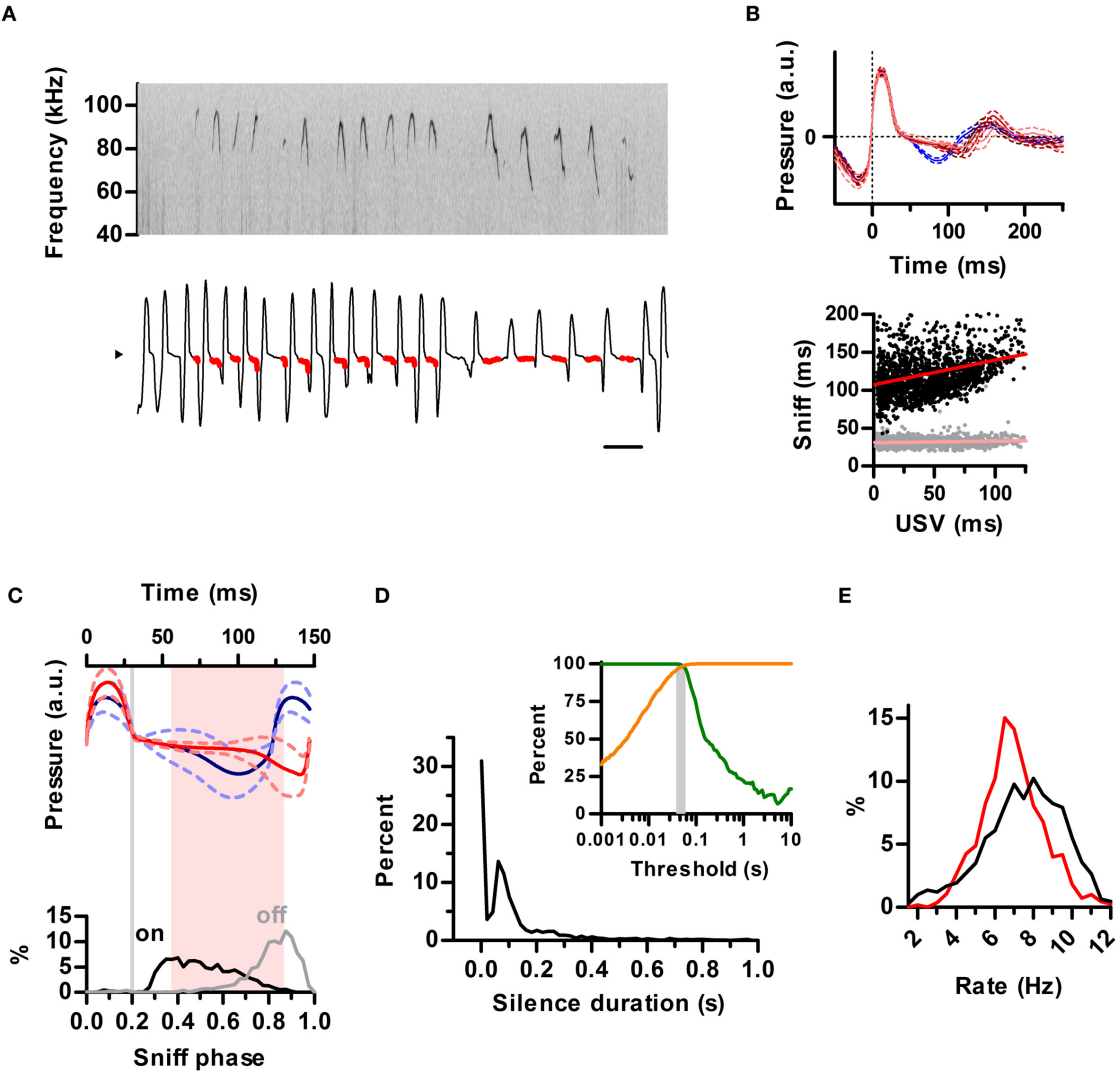
**Structuring of mouse ultrasonic vocalizations by sniffing. (A)** Detailed view of ultrasonic vocalizations (top; sonogram) and respiration (bottom) for a mouse. Scale bar: 250 ms. Compare to Figure 1C. **(B)** Top: average waveforms for silent sniff cycles (blue) or cycles simultaneous to the emission of ultrasonic vocalizations of increasing duration (reds; vocal sniffs) for a mouse. Data was binned by ultrasound duration (mean durations: 14, 42, and 81 ms). Traces are aligned to inhalation onset (dotted line). Compare with Figure 3A. Bottom: Inhalation (gray) and exhalation (black) durations for individual vocal sniff cycles vs. vocalization duration across mice. Lines: linear regressions; Slope = 0.32 (exh) and 0.02 (inh), *R*^2^ = 0.08 (exh) and 0.01 (inh). Compare with Figure 3B. **(C)** Top: mean sniff waveforms from silent (blue) or vocal (red) sniffs across mice. All waveforms were warped to align at three points: onsets of inhalation and exhalation and the end of exhalation. Bottom: Distribution of ultrasound onset (black) and offset (gray) phases in the vocal sniffs. Inhalation onset: phase = 0, exhalation onset: phase = 0.2, end of exhalation: phase = 1. Gray line: exhalation onset. Time between most frequent vocalization onset and offset marked in pink. Compare with Figure 4B. **(D)** Segmentation of calls as a function of silence duration threshold in mice. Orange: percentage of segmented calls that do not share a sniff cycle with other calls. Green: percentage of calls that do not span more than one sniff cycle. The gray area shows the range of silence duration thresholds that effectively segment over 95% of calls by sniff cycles (40–60 ms). Compare with Figure 5C. **(E)** Blue: distribution of silent sniff rates. Red: distribution of instant call rates for calls made on consecutive sniffs.

We next extended our analysis to the ultrasonic vocalizations of the laboratory mouse (*Mus musculus*). We simultaneously recorded vocal output with intranasal pressure in male CBA/CaJ adults (*N* = 2) during encounters with a female. Previous studies have concluded females rarely, if ever, emit USVs during mating so detected ultrasonic calls can be assigned to the male partner (White et al., 1998). Indeed, all calls detected from our recordings matched the breathing pattern of the male (Figure 7A). The sniff cycles of mice differed from that of rats in that even for silent sniffs, inhalations were followed by a brief period of constant low relative intranasal pressure before going into full exhalation (Figure 7A), whereas in the rat this pattern was strongly indicative of USVs (see Figures 1C, 3A, 4A,B and Figure S2). As in the rats, the emission of USVs significantly prolonged the sniff cycle, with a positive correlation between exhalation duration and the duration of USV (Figure 7B). The slope of this relationship was less pronounced (compare Figures 7B, 3A,B). Nonetheless, the locking of the ultrasound production to the phase of the sniff cycle was comparable to that found for rats, with USVs starting after the end of the inhalation and ending prior to the peak of the exhalation (Figure 7C).

The temporal properties of ultrasonic calls in the mouse were qualitatively similar to the rat. Silence durations of at least 40–60 ms segmented ultrasonic output into calls (mean duration = 46 ms) occurring within a single sniff (Figure 7D). The distribution of instantaneous rates of calls produced on consecutive sniffs peaked at 6.5 Hz whereas instantaneous rates of silent sniffs peaked at 8 Hz (Figure 7E). This shift is a direct result of prolongation of exhalations by calls, as also observed for the rats.

### Call bouts are different in rats and mice

**Figure 8:**
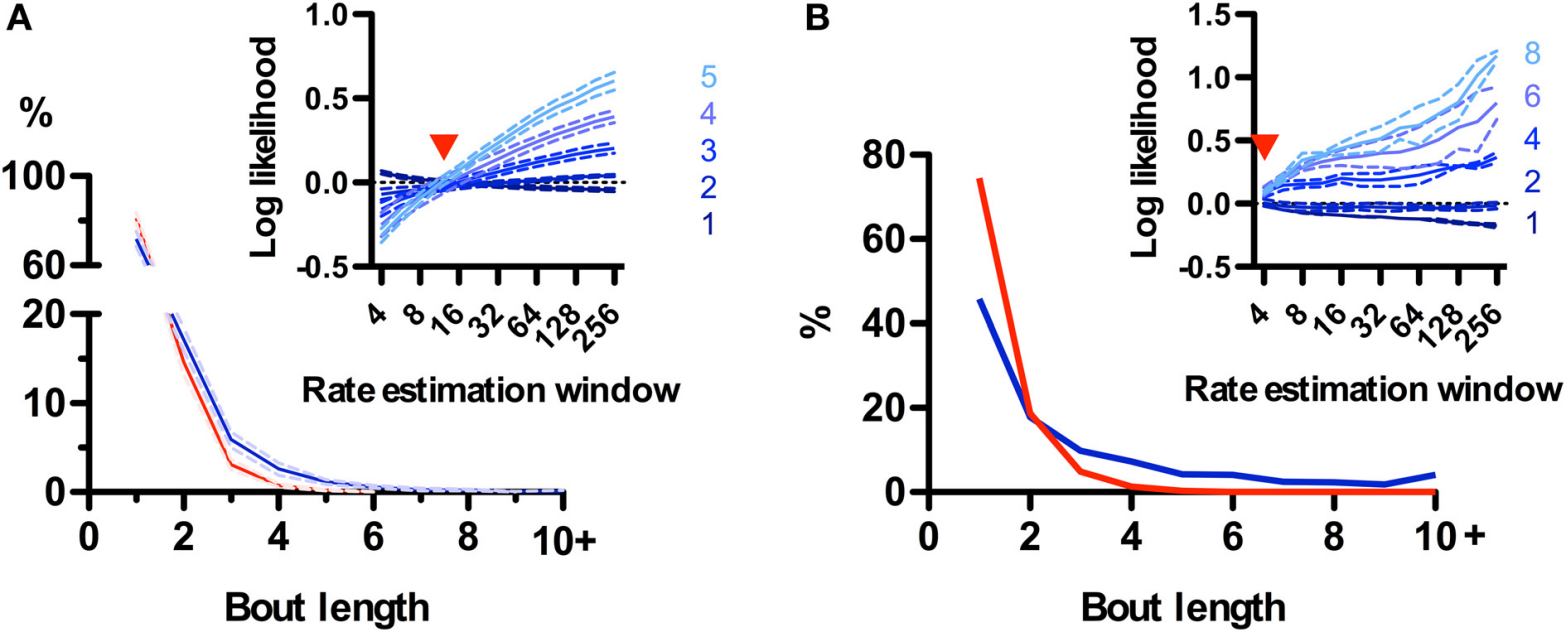
**Call bouts are different in rats and mice. (A)** Probability of observing rat call bouts of a given length (i.e., the number of consecutive sniffs with calls). Blue: real measured data. Red: surrogate data constructed assuming constant vocalization rate (see Materials and Methods). Inset: Comparison of measured bout length probabilities to a family of surrogate models with varying rate estimation windows (4–256 sniffs; x-axis; see Materials and Methods). Y-axis: log likelihood ratio between measured and surrogate bout length probabilities (for bout length 1–5). Positive values indicate that bouts of a given length are more likely in real vs. surrogate data. Red arrowhead: surrogate model with a rate estimation window of width 12 sniffs matches real data for all bout lengths (log-likelihood ≅ 0). Panels show mean ± s.e.m.; *N* = 5 rats. **(B)** Same analysis as in A for mice. Note lower probability of bout length = 1 for mice (46%) than for rats (72%). Surrogate data with a 4-sniff rate estimation window approximates observed bout distribution in mice, compared with 12-sniff window for rats.

While rodent USVs appear to cluster in time (Nyby and Whitney, 1978; Brudzynski and Pniak, 2002), it is not clear whether the call “bout” is a fundamental unit of their vocal production. Alternatively, calls could appear to be grouped in time simply because of continuous fluctuations in call rate (Nawrot, 2010). We took advantage of the natural segmentation provided by the sniff cycle to explore this in rats and mice. We defined a bout as a series of calls emitted on consecutive sniffs and asked whether their occurrence was a statistically significant event. At first glance, no strong tendency for emitting bouts was observed for rats, as the distribution of bout lengths decayed monotonically with 72 ± 4% (*N* = 5 rats) composed of a single call and only 2.5 ± 0.7% containing 5 or more calls (Figure 8A). To test for structure in the vocal production we compared this distribution with a random model where rats have a constant probability of emitting a call on each sniff given by their mean call rate (see Materials and Methods). Bouts of 3 or more calls occurred more frequently than chance, while isolated calls were in fact less probable (Figure 8A). However, when comparing with a family of random models that account for call rate variations, the grouping of calls into bouts matched models where calls are randomly emitted with a probability fluctuating with a temporal resolution of 1–2 s (Figure 8A, inset). This analysis suggests that call bouts defined in this way are not a fundamental feature of rat vocal production but rather reflect fast modulations in their behavioral state. Mouse calls were emitted in strikingly longer bouts than for those of rats, with only ~45% of them composed of a single call and ~20% containing 5 calls or more (Figure 8B). This high structuring could not be accounted for by random models with slow call rate fluctuations (Figure 8B, inset), suggesting mice USVs are indeed preferentially grouped into bouts.

## Discussion

By examining long periods of simultaneously recorded respiration and ultrasonic vocalization patterns we found a profound relationship between these two behaviors across timescales. Overall, vocal production is largely restricted to periods of active sniffing. During these periods, both sniffs and calls are periodic at theta frequencies (6–8 Hz). USVs are not, however, a byproduct of olfactory behavior as rats can sniff fast without vocalizing. Calls are produced exclusively during exhalations and prolong sniffs causing an instantaneous reduction in sniff rate. Most calls are, however, brief, producing only a modest drop in sniff rate from 8 to 6 Hz. In this way, the rate of ongoing sniffing effectively imparts its theta rhythmicity onto calls.

Though it is commonplace in the field to talk about rodent “calls,” a proper delineation of the term is missing. Segmenting a stream of vocal output into meaningful units is an important first step in any semantic or syntactic study. The working hypothesis behind defining animal “calls” is that there are a finite number of distinct motor plans for the production of vocalizations which could differentially correlate with the emitter's physiological or behavioral state and the receiver's responses. Segmentation of the produced sound by this underlying structure results in a more compact description of the vocal repertoire and aids in the understanding of vocal communication systems. Animal vocalization is usually broken up in calls at spectrotemporal discontinuities, but the choice of parameters is not trivial. We propose a physiologically grounded segmentation strategy such that a call is defined as the ultrasound emitted during a single exhalation. We further show this rule can be accurately implemented without recording respiration by choosing a silence duration threshold between 20 and 60 ms for rats and 40–60 ms for mice. Of those studies where the segmentation method is reported, some used silence durations within or close to these ranges (Liu et al., 2003; Holy and Guo, 2005; Wright et al., 2010) while others used thresholds too short to match the sniffing structure (Sewell, 1970; Takahashi et al., 2010).

**Figure 9:**
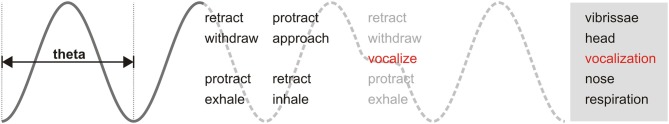
**Theta-linked orofacial behaviors in rodents**. Periodic motor actions during active behavioral states are coordinated in phase along a theta frequency rhythm. When vocalizations occur (red), they are inserted immediately after the end of inhalation. Adapted after Welker (1964) and Kepecs et al. (2006).

Welker's detailed examination of rat behavior demonstrated the phasic relationship between sniffing, whisking, and head movement. During active periods, these behaviors are produced in cycles coherent at theta frequency (Welker, 1964; Deschênes et al., 2012; Moore et al., 2013; Ranade et al., 2013). This shared oscillatory patterning has been proposed to be relevant for information exchange between brain areas (Kay, 2005; Kepecs et al., 2006). Our results add the emission of ultrasonic vocalizations to the family of orofacial behaviors with theta rhythmicity observed in rodents (Figure 9). As such, the detailed properties of USVs are not independent but bounded by this rhythmic frame. Any research into the neural or broader behavioral correlates of any such motor behaviors would thus benefit from considering the broad context of the others to identify any individual contributions and interactions (Assini et al., 2013; Moore et al., 2014). During ultrasound production, motoneurons in the nucleus ambiguus control larynx geometry via activation of specific muscles (Yajima and Hayashi, 1983; Riede, 2011). The observed phase locking of vocalizations with the sniff cycle suggests a precise coordination between activity in this motoneuron pool and the brainstem nuclei responsible for orchestrating the respiratory rhythm (Moore et al., 2014). The mechanistic links posited by our observations should be confirmed by experimental manipulation of activity in these nuclei, as is being done for dissecting the interactions between the sniffing and whisking rhythms (Moore et al., 2013). Our results show that constriction of the larynx associated with ultrasound production is associated with a delay in the onset of the following respiratory cycle, similar to that observed for swallowing (McFarland and Lund, 1993). USVs are natural and frequent perturbations of the sniffing cycle. Understanding how they affect (and are affected by) the instantaneous phase of other orofacial rhythms like whisking and head movements could aid in understanding the hierarchical organization of their associated motor nuclei. Of particular interest is the coordination of ultrasonic vocalization with active whisking, as it is likely that both are simultaneously acting as rhythmic communication signals during close distance social interactions (Wolfe et al., 2011).

The rate of respiration is strongly correlated with the behavioral state of the animal (Welker, 1964; Hegoburu et al., 2011). We show that calls carry detailed information about sniff dynamics at both slow and fast timescales. At slow scales, the co-occurrence of high rates of 50 kHz USVs and fast sniffing could reflect their common drive by the ascending dopaminergic system (Costall and Naylor, 1975; Brudzynski, 2007). Given this link, 50 kHz USVs could preferentially promote social contact in individuals in positively aroused, exploratory states. At faster time scales, calls group together in time resulting in bouts where calls are emitted in consecutive sniffs. We found that the statistics of rat call bouts do not support their status as a fundamental unit of vocal production, but rather appear secondary to changes in the drive to produce calls on the timescale of 1–2 s. In contrast, mouse calls are organized into longer bouts that cannot be accounted for by slow rate fluctuations, in agreement with a proposed song-like production (Holy and Guo, 2005). Call instant rates within bouts are centered on theta, with their precise value closely reflecting the underlying sniffing rate. Thus, the instantaneous call rate could transmit detailed information about the ongoing sniffing rate of the emitter, which is intimately linked with behavioral state. Interestingly, sounds presented at these rates are privileged in their processing by the auditory system of rats. During development, the auditory cortex selectively enhances the representation of sounds presented within theta band ~7 Hz (Kim and Bao, 2009), suggesting that theta patterning is important for the learning of species specific vocalizations. In adults, auditory responses to sounds are heavily attenuated at presentation rates above 10 Hz (Kilgard and Merzenich, 1998), which corresponds to the upper limit of our observed distribution of instantaneous call rates. Thus, the auditory system of rodents may be preferentially tuned to the sniff-driven dynamics of conspecific vocalizations.

Other mammalian orofacial communication signals are temporally structured at theta frequencies, such as marmoset twitter calls (Wang et al., 1995), macaque lip-smacking (Ghazanfar et al., 2010) and human speech (Chandrasekaran et al., 2009). Specific disruption of this rhythmicity results in impaired intelligibility (Saberi and Perrott, 1999; Ghitza and Greenberg, 2009; Ghazanfar et al., 2013) and cortical oscillations at matching frequencies are proposed to play a role in their selective perception (Giraud and Poeppel, 2012). Whether theta rhythms in primate and rodent social signals are evolutionarily linked and whether emission and perception of all of them are linked to cortical theta oscillations remains unknown.

### Conflict of interest statement

The authors declare that the research was conducted in the absence of any commercial or financial relationships that could be construed as a potential conflict of interest.
