# Supplementary material for: Rodent ultrasonic vocalizations are bound to active sniffing behavior
Source: Front Behav Neurosci. 2014 Nov 18;8:399. doi: 10.3389/fnbeh.2014.00399 (PMC4235378; doi:10.3389/fnbeh.2014.00399)
Supplement: Supplementary file 1 [file Presentation1.PDF]

# *Supplementary Material*

# Rodent ultrasonic vocalizations are bound to active sniffing behavior

**Yevgeniy Sirotin[1], Martín Elias Costa[2] & Diego A. Laplagne[1,3]**

[1]Shelby White and Leon Levy Center for Brain, Mind and Behavior, The Rockefeller University, New York, NY.
[2]Integrative Neuroscience Lab, Department of Physics, University of Buenos Aires, Buenos Aires, Argentina.
[3]Brain Institute, Federal University of Rio Grande do Norte, Natal, RN, Brazil.

*** Correspondence:** Diego A. Laplagne, Brain Institute, Federal University of Rio Grande do Norte, Av. Nascimento de Castro 2155, Natal, RN, 59056-450, Brazil.
dlaplagne@neuro.ufrn.edu

## 1. Supplementary Figures

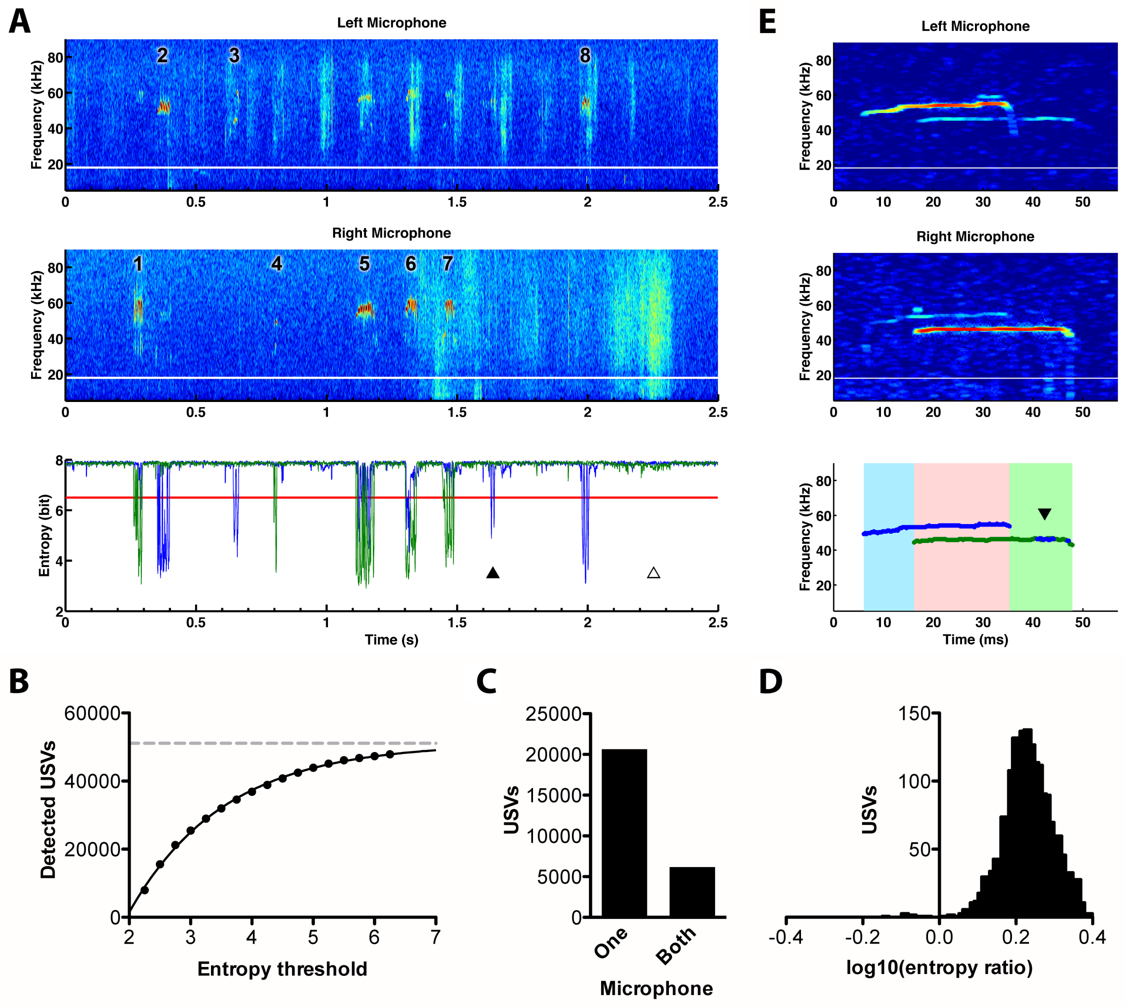


**Supplementary Figure 1. Detection and assignment of ultrasonic vocalizations from our social arena.** **A.** Example recording with one rat on each side of the arena (see Figure 1A). Top and middle panels show sonograms for the left and right microphones. Bottom panel shows instantaneous entropy (blue: left mic; green: right mic) at frequencies above 18 kHz (white line on sonograms). USVs 2, 3, 8 and 1, 4, 7 were readily assigned to the rats on the left and right respectively as they crossed the entropy threshold (red line) on only their respective microphone. Calls 5 and 6 were detected on both microphones and assigned to the rat on the right based on its lower entropy values. Low-entropy segments of under 3 ms are rejected (arrowhead). Broadband bedding noises (open arrowhead) have little effect on the entropy. **B.** Total number of detected USVs in 31 recordings as a function of chosen entropy threshold. 47866 calls are detected at the value of 6.25, used throughout this work. The exponential fit reaches plateau at 51095, suggesting our analysis detects 94% of the emitted calls. **C, D.** Recordings with only one rat (other side of the arena empty) can be used to assess the accuracy of the call assignment, as all of them should correspond to the microphone on the rat's side. In 11 recordings (26815 total calls), 77% of calls crossed entropy threshold only on the microphone over the rat (C). Of those detected in both, the entropy on the correct microphone is lower enough to that on the opposing one to unambiguously assign it (D, log(entropy ratio) >> 0). Overall, 99.8 ± 0.1 % of calls were properly assigned in each recording to the side of the arena where the rat was. **E.** Example of USVs simultaneously emitted by two rats as picked up by both microphones (top and middle panels). Note both USVs are observed in both sonograms, although with different intensity. The frequency with maximum power is extracted from both sonograms and compared side by side (bottom). Time segments of over 3 ms with frequency differences > 1 kHz are considered to have superimposed USVs and assigned to both rats (light red shading). Times assigned to just one rat are shaded light blue and green. Note part of the same USV is detected at both microphones but only assigned to the one with lower entropy (arrowhead).

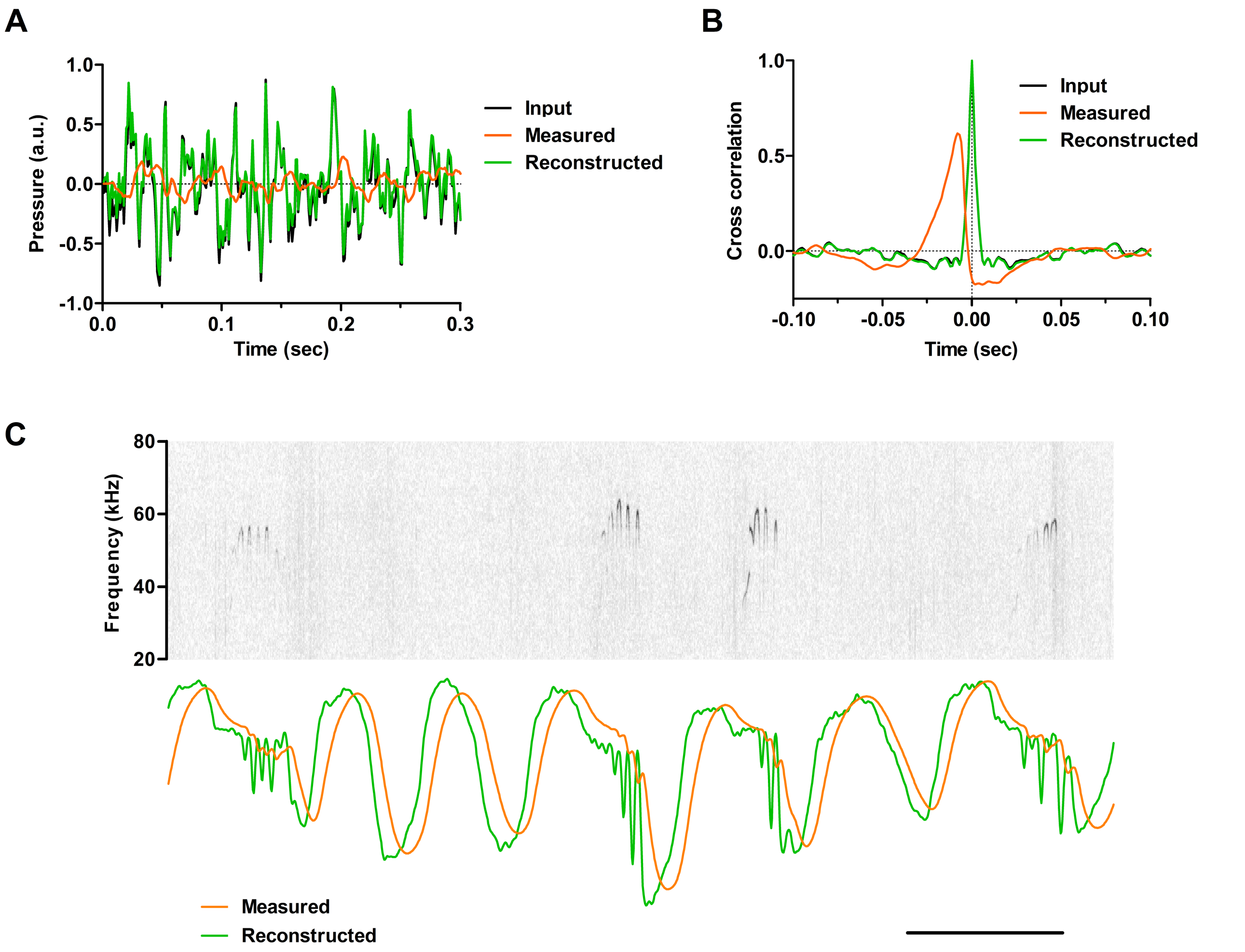


**Supplementary Figure 2. Correction of pressure signals for tubing distortion. A.** Black: example of broadband pressure noise (0-11 kHz maximum) measured directly at the output of the transducer. Orange: same pressure signal measured at the output of a Teflon tubing used for intranasal pressure recordings. Green: same signal after reconstruction by deconvolution. Deconvolved traces matched precisely the signals measured without the tubing. **B.** Correlation functions between the noise signal and itself (black), after distortion by the tubing (orange) and after reconstruction (green). **C.** Top: Example sonogram measured during and experiment. Bottom: intranasal pressure as measured out of the tubing (orange) and after correcting through deconvolution (green). Note how the time delay is corrected and high frequency details are recovered. Scale bar: 200 ms.

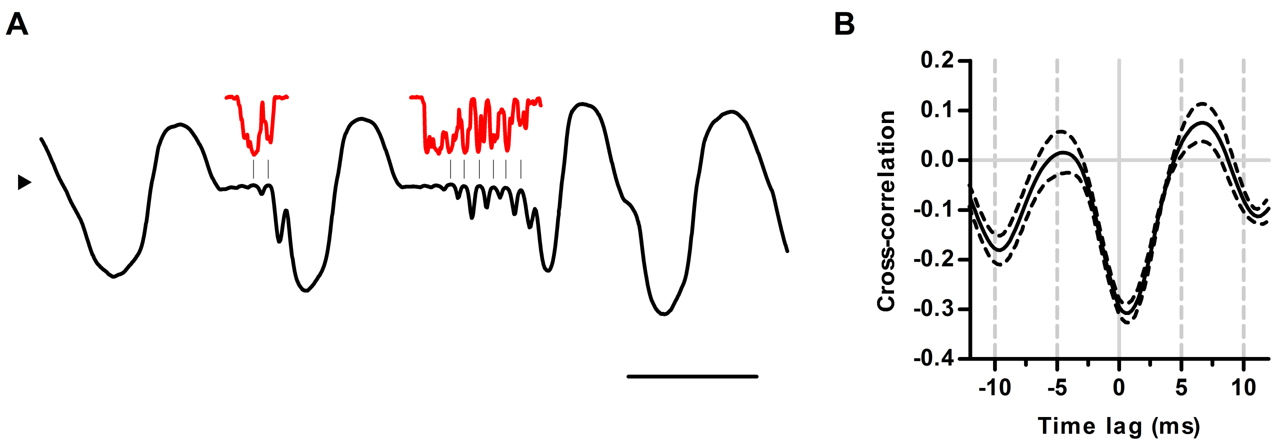


**Supplementary Figure 3. Millisecond alignment between intranasal pressure and ultrasound production. A.** Example intranasal pressure signals measured using a head-mounted pressure transducer (black) and aligned ultrasound entropy traces at times with detected USVs (red). Vertical lines highlight alignment between low sound entropy (high USV power) and low intranasal pressure. **B.** Cross-correlation function between the pressure and ultrasound signals during USV emission as a function of time lag. Error bars are standard deviation across rats.

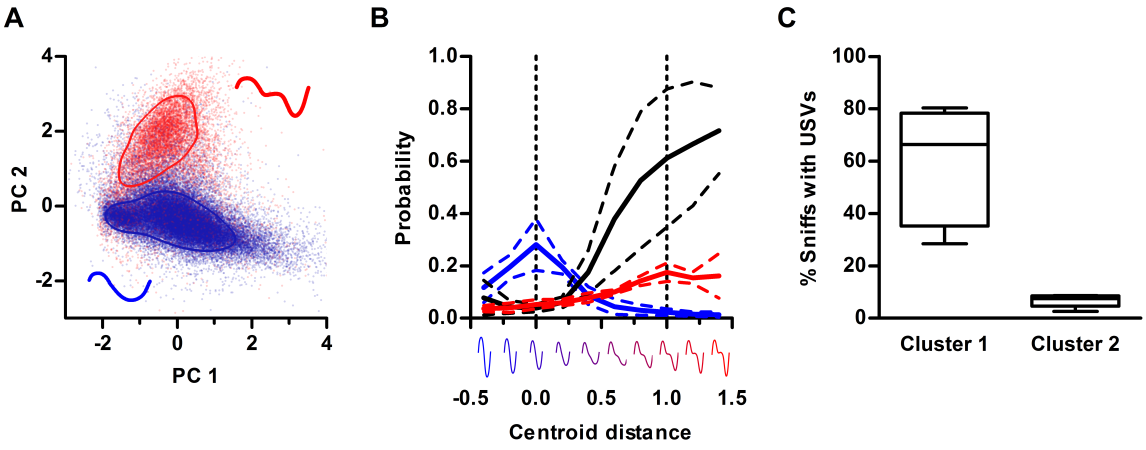


**Supplementary Figure 4. Sniff shape in the rat can predict the presence of a USV.**
**A.** Individual sniffs recorded for one rat projected on the first two principal components obtained from the distribution of sniff shapes during exhalation. Red and blue contours correspond to two clusters obtained using k-means clustering of the data in these principal components. Red points are sniffs with USVs and blue points are silent sniffs. **B**. Probability of a sniff being vocal (black trace) as a function of position between centroids of the two sniff clusters. Red and blue traces are the distributions of silent (blue) and vocal (red) sniffs computed along the inter-cluster trajectory. Distance is normalized so that the centroid of the blue cluster = 0 and the centroid of the red cluster = 1. Shapes below the y-axis show the shape of the average warped sniffs obtained long the inter-cluster trajectory. Error bars are standard deviation across rats. **C**. Box plots showing proportion of sniffs with USVs in each cluster across rats.
